# Deficiency of MecA in *Streptococcus mutans* Causes Major Defects in Cell Envelope Biogenesis, Cell Division, and Biofilm Formation

**DOI:** 10.3389/fmicb.2018.02130

**Published:** 2018-09-11

**Authors:** Arpan De, Ashton N. Jorgensen, Wandy L. Beatty, Jose Lemos, Zezhang T. Wen

**Affiliations:** ^1^Department of Comprehensive Dentistry and Biomaterials, University of Florida, Gainesville, FL, United States; ^2^Center of Oral and Craniofacial Biology, University of Florida, Gainesville, FL, United States; ^3^Department of Microbiology, Immunology and Parasitology, Louisiana State University Health Sciences Center, New Orleans, LA, United States;; ^4^Department of Molecular Microbiology, Washington University in St. Louis, St. Louis, MO, United States; ^5^Department of Oral Biology, University of Florida, Gainesville, FL, United States

**Keywords:** *Streptococcus mutans*, MecA, stress tolerance response, biofilms, cell envelope biogenesis, Clp protease complex

## Abstract

MecA is an adaptor protein that guides the ClpC/P-mediated proteolysis. A *S. mutans* MecA-deficient mutant was constructed by double-crossover allelic exchange and analyzed for the effects of such a deficiency on cell biology and biofilm formation. Unlike the wild-type, UA159, the *mecA* mutant, TW416, formed mucoid and smooth colonies, severely clumped in broth and had a reduced growth rate. Transmission electron microscopy analysis revealed that TW416 grows primarily in chains of giant “swollen” cells with multiple asymmetric septa, unlike the coccoid form of UA159. As compared to UA159, biofilm formation by TW416 was significantly reduced regardless of the carbohydrate sources used for growth (*P* < 0.001). Western blot analysis of TW416 whole cell lysates showed a reduced expression of the glucosyltransferase GtfC and GtfB, as well as the P1 and WapA adhesins providing an explanation for the defective biofilm formation of TW416. When analyzed by a colorimetric assay, the cell wall phosphate of the mutant murein sacculi was almost 20-fold lower than the parent strain (*P* < 0.001). Interestingly, however, when analyzed using immunoblotting of the murein sacculi preps with UA159 whole cell antiserum as a probe, TW416 was shown to possess significantly higher signal intensity as compared to the wild-type. There is also evidence that MecA in *S. mutans* is more than an adaptor protein, although how it modulates the bacterial pathophysiology, including cell envelope biogenesis, cell division, and biofilm formation awaits further investigation.

## Introduction

*Streptococcus mutans*, a common inhabitant of the tooth surface, is considered as a primary causative agent of human dental caries. *S. mutans* possesses multiple mechanisms to colonize the tooth surface and accumulate in the plaque biofilms, which include cell surface adhesin (SpaP), glucosyltransferases (GtfB, -C, and -D) and glucan binding proteins (Gbps) (Bowen and Koo, 2011). The Gtf enzymes, their adhesive extracellular polysaccharide products and the Gbps, along with other polymeric substances such as extracellular deoxyribonucleic acids and fibrillar protein aggregates (also amyloids), play essential roles in *S. mutans’* establishment and accumulation on the tooth surfaces, central to its cariogenicity (Koo et al., 2010; Besingi et al., 2017). *S. mutans* biofilm formation is regulated in response to various environmental cues and the presence of other bacterial species (Burne et al., 2011; De et al., 2017). Multiple two-component signal transduction systems, molecular chaperones, quorum sensing, and factors such as biofilm regulatory protein BrpA and autolysin AtlA are shown to significantly influence *S. mutans* cellular biology and biofilm formation (Burne et al., 2011; De et al., 2017).

The oral cavity is a dynamic environment, where oral bacteria often encounter frequent and rapid fluctuations in pH, temperature, osmolarity, and the concentrations of various antimicrobial agents, such as hydrogen peroxide, sodium lauryl sulfate, and chlorhexidine (Bitoun et al., 2012). One of the consequences of exposure to environmental stresses is the accumulation of abnormal proteins due to increased errors in transcription and translation (Lemos and Burne, 2008). Molecular chaperones, such as DnaK, DnaJ, and GroEL, are required for proper folding and assembly of those aberrant proteins (Lemos et al., 2007). Proteases, such as Clp proteases, are involved in the degradation of the proteins, not only under stress conditions, but also under normal growth conditions (Lemos and Burne, 2002; Chattoraj et al., 2010; Kajfasz et al., 2011). It is central for bacterial viability, persistence and growth to maintain protein homeostasis by stabilizing proteins that perform essential functions and by refolding or degrading misfolded or aberrant proteins (Lemos et al., 2007).

The bacterial Clp proteolytic complex is structurally similar to the eukaryotic 20S proteasomal complex and is comprised of an AAA^+^ ATPase subunit, such as ClpE, ClpX, or ClpC, and a proteolytic component known as ClpP (caseinolytic protease) (Chattoraj et al., 2010; Kajfasz et al., 2011). The AAA^+^ ATPases typically recognize, denature, and translocate protein substrates into the proteolytic core of the ClpP peptidase for subsequent digestion (Frees et al., 2007). Often adaptor proteins, such as MecA and SspB, which enhance and diversify the substrate spectra of their cognate AAA^+^ ATPases, modulate the function of the Clp-proteolytic complexes. Wide-spread among low-GC Gram-positive bacteria, adaptor protein MecA is required for functional ClpC complex formation or assembly of the ClpCP degradation machine (Turgay et al., 1998; Persuh et al., 1999; Liu et al., 2013), which in *B. subtilis* is well documented to play a key role in regulation of growth, sporulation, and genetic competence (Turgay et al., 1998; Persuh et al., 1999).

In streptococci, MecA acts as a negative regulator of alternative sigma factor SigX, also ComX, a key transcriptional regulator for activation of late competence genes (Luo et al., 2003; Aspiras et al., 2004; Mashburn-Warren et al., 2010; Boutry et al., 2012; Ahn et al., 2014). Like *B. subtilis*, MecA interacts with SigX and ClpC forming a ternary complex, SigX-MecA-ClpC, recognizing and targeting SigX for proteolytic degradation by ClpP, thus modulating the expression of genes required for development of genetic competence (Tian et al., 2013; Dong et al., 2014; Wahl et al., 2014). In *S. mutans*, deficiency of MecA, ClpC, or ClpP was shown to result in cellular accumulation of SigX and a prolonged competence state, while over expression of MecA enhances proteolysis of SigX and accelerates the escape from competence (Tian et al., 2013; Dong et al., 2014).

**FIGURE 1:**
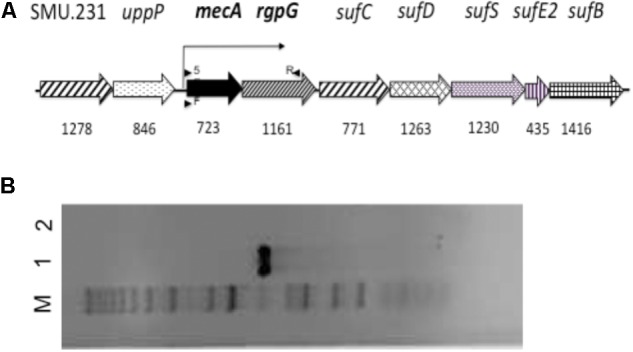
The schematic diagram of the *mecA* flanking region **(A)** and RT-PCR analysis **(B)**. **(A)** Schematic diagram of the flanking regions of *mecA* in the genome of *S. mutans* UA159. Numbers underneath represent the size of the respective genes. Arrows above and underneath indicate the location and orientation of the primers used for reverse transcription (5) and cDNA amplification (F and R). **(B)** RT-PCR analysis of the *mecA*/*rgpG* cluster. cDNA generated from reverse transcription using total RNA extract of *S. mutans* was PCR amplified using primer set PmecARgpG (lane 1), which shows a single amplicon indicative of co-transcription of *mecA* and *rgpG*. Negative control (lane 2) with no reverse transcriptase shows no DNA amplification. M, for molecular marker.

In *S. mutans* UA159, *mecA* (SMU.245) and its immediate downstream *rgpG* are predicted as a polycistronic operon (**Figure 1A**), where *rgpG* encodes the first enzyme of the biosynthesis pathway of rhamnose-glucose polysaccharide (RGP) polymers, a major cell wall associated antigen in oral streptococci (Yamashita et al., 1999; De et al., 2017). Herein, we have provided direct evidence that *mecA* and *rgpG* are co-transcribed. Relative to the parent strain, deficiency of MecA led to major alterations in colony and cell morphology, severe defects in cell division and biofilm formation. These results suggest that other than competence development, MecA also plays a significant role in *S. mutans* cell envelope biogenesis and regulation of virulence traits independent of the ClpC/P regulated proteolysis.

## Materials and Methods

### Bacterial Strains and Growth Conditions

All the bacterial strains used in this study were grown in Brain Heart Infusion (BHI) (Difco Lab.) at 37°C, 5% CO_2_. When required, kanamycin (1 mg/mL) and/ or spectinomycin (1 mg/mL) were added to the medium. For growth studies at different conditions, cells grown until mid-exponential phase (OD_600_ = 0.5–0.6) were appropriately diluted in BHI medium (1:100) and growth was continuously recorded by Bioscreen C (Oy Growth curves) at 37°C with and without mineral oil overlay (Bitoun et al., 2013). Growth studies in acidic and oxidizing environment were assessed using BHI adjusted to pH 6.0 and in the presence of methyl viologen (Sigma, 12.5 mM), respectively.

### Construction of the Mutant and Complement Strain

The *mec*A mutant was constructed by PCR ligation mutagenesis method as described previously (Lau et al., 2002). Briefly, flanking regions of *mec*A were amplified using high fidelity DNA polymerase (Q5, NEB) with gene specific primer sets NR5 and NR3 (**Table 1**). The resulting amplicons were digested with XbaI and ligated to a non-polar spectinomycin resistance element (*aadA*) that was digested with the same enzymes. The resulting ligation mix was used to transform *S. mutans* UA159 in the presence of CSP and the double-crossover mutants (TW416) were selected on BHI agar containing spectinomycin. A kanamycin resistant *mecA* mutant was also made using a non-polar kanamycin resistance marker (Hossain and Biswas, 2012) replacing the coding sequence using similar strategies as described above. The *mecA* deletion and replacement were confirmed by PCR and Sanger sequencing using gene specific primers. To construct the complement strain, *mecA* along with its cognate promoter region was amplified with gene-specific primers (**Table 1**) and cloned into integration vector pBGK(3) (De et al., 2017). Subsequently, the resulting construct, pBGK(3):P*mec*A, was transformed into TW416, and the transformants (TW416C) were selected on BHI agar plates containing spectinomycin and kanamycin.

**Table 1:** Primers used in this study^∗^

Name	Forward (5′→3′)	Reverse (5′→3′)	Application
NR5	Agtactgggcatttgattttagtg	Tctttcttctagatcttccatactgatg	5′ fragment for *mecA* mutation
NR3	agtctagagcttatcacatgactgttc	Tgatgtaagtgcatattatcagcttc	3′fragment for *mecA* mutation
mecAc	tcgagtaacaccaaggaatgatc	Tccagcacaaagtgaacgtaatc	verification of *mecA* deletion
mecAsc	Tttaatgagctgaactccatgttc	Ataactactcgtatgaggtaaagac	*mecA* sequencing confirmation
*mecAsc3b*		*Tctaaatcttccatactgatggtg*	*mecA sequencing confirmation*
mecAcp	tactgggctagcgattttagtggaag	Actaaaggagctcaaacaagtgaagtc	*mecA* complementation
PmecA5F (5)	Acccgaaaagaacatattac		reverse transcription
PmecARgpG	F: tgggcttttcttgtataatg	R: cccacaaaatcgttaagaag	cDNA amplification

*^∗^Underlined sequences indicate restriction sites used for cloning*.

### Acid and Hydrogen Peroxide Killing Assays

The effects of MecA-deficiency on the ability of *S. mutans* to withstand acid and oxidative stresses were assessed by using acid killing and hydrogen peroxide challenge assays as described elsewhere (Wen and Burne, 2004). Briefly, for these assays, planktonic cultures of *S. mutans* strains were grown in BHI until mid-exponential phase (OD_600nm_ = 0.3–0.4) and then subjected to acid and hydrogen peroxide killing (Wen and Burne, 2004).

### TEM Analysis

*Streptococcus mutans* strains were grown in BHI until OD_600_≅0.4, harvested by centrifugation (3220 ×*g* at 4^o^C for 15 min), washed once with phosphate buffered saline (PBS, 20 mM, and pH 7.0) and then fixed in 2% paraformaldehyde/2.5% glutaraldehyde (Polysciences, Warrington, PA, United States) in PBS for 1 h at room temperature. Cells were then washed in PBS, fixed in 1% osmium tetroxide (Polysciences) for 1 h and then rinsed in water prior to en bloc staining for 1 h with 1% aqueous uranyl acetate (Ted Pella Inc., Redding, CA, United States). The cells were then washed in water and dehydrated in a graded series of ethanol followed by embedding in Eponate 12 resin (Ted Pella Inc., Redding CA, United States). Sections of 90–100 nm were prepared, stained with uranyl acetate and lead citrate, and viewed under a JEOL 1200 EX transmission electron microscope (JEOL United States Inc., Peabody, MA, United States) equipped with an AMT eight megapixel digital camera and AMT Image Capture Engine V602 software (Advanced Microscopy Techniques, Woburn, MA, United States).

### Biofilm Analysis

The sessile growth of *S. mutans* strains were determined in modified biofilm medium with glucose (20 mM and BMG), sucrose (20 mM and BMS) or glucose and sucrose (18 mM and 2 mM, respectively, BMGS) as described previously (Loo et al., 2000; Li et al., 2002). In brief, cells grown until mid-exponential phase in BHI were diluted in appropriate biofilm medium and grown in 96 well microtiter plate (Costar Inc., United States). After 24 h of incubation, the biofilms were stained in 0.1% crystal violet, bound dye was extracted using an acetone-ethanol mixture (1:4), and absorbance was measured using in Synergy II plate reader (BioTek) (Wen and Burne, 2002). The texture and structure properties of the biofilms were evaluated on hydroxylapatite (HA) disks grown in BMGS. After 24 h of incubation, the biofilms were stained using *Bac*Light Live/Dead staining kit (Invitrogen), followed by its optical dissection in a Confocal Laser Scanning Microscope (Olympus, Fluoview BX61) using a 60× water immersion objective lens. For each strain, image stacks were acquired from at least three different regions on the HA disk, and the image stacks were processed in SLIDEBOOK 5.0 (Olympus) and further analyzed using COMSTAT 2.0 (Heydorn et al., 2000). For SEM analysis, biofilms were fixed using 2.5% glutaraldehyde (Polysciences, Warrington, PA, United States) in buffered saline (PBS, pH 7.4), dehydrated using increasing concentrations of ethanol, critical point dried, carbon coated and analyzed using a field emission-scanning electron microscope (Hitachi Ltd., Tokyo, Japan) under 5 kV accelerate voltage (Wen and Burne, 2004).

### Reverse-Transcription Analysis and Reporter Fusion Assays

For RT-PCR, total RNA was extracted from *S. mutans* cells grown in BHI until early exponential phase (OD_600nm_ ≅ 0.3) using hot phenol (Wen et al., 2006). It was then treated with DNase I (Ambion) and retrieved using RNeasy purification kit (Qiagen, Inc.). Reverse-transcription and PCR were then used to evaluate the co-transcription using gene specific primers (**Table 1** and **Figure 1**). To examine the potential role of MecA in its own regulation and regulation of *rgp*G and the *rgpA/F* operon, the cognate promoter of the respective gene(s) were amplified using gene-specific primers (**Table 1**), and following proper restriction enzyme digestions, fused in front of a promoterless luciferase gene (*luc*) in the integration vector pFW11-*luc* (Podbielski et al., 1999). Following verification of the inserted sequence accuracy by Sanger sequencing, the resulting fusions were transformed into the *mecA* mutant and UA159. The impact of MecA-deficiency on the expression of targeted gene(s) was analyzed by luciferase assay by following the protocol of Podbielski (Podbielski et al., 1999; Liao et al., 2017).

### Antimicrobial Susceptibility Tests

The susceptibility of *S. mutans* strains to different antimicrobial agents targeting cell envelope was assessed by microtiter plate-based assay following protocol as described elsewhere (Bitoun et al., 2012; Bitoun et al., 2013). The tested antibiotics included lipid II inhibitors (vancomycin, bacitracin and nisin) and non-lipid II inhibitors (penicillin G and D-cycloserine), while the compounds affecting cell membrane included SDS and Chlorhexidine.

### Cell Lysate Preparation and Western Blot Analysis

*Streptococcus mutans* strains were grown until early exponential phase (OD_600nm_ = 0.3) in BHI with appropriate antibiotics, washed once in sterile PBS, and then lysed in 100 mM Triethylammonium bicarbonate (TEAB) buffer containing 1% SDS and protease inhibitors (Sigma) using a Bead beater for 30 s, thrice, with an intermittent transfer on ice for 1 min (Bitoun et al., 2012). The clarified lysate was collected by centrifugation at 10,000 ×*g*, 4°C for 10 min. The total protein concentration was measured using BCA kit (Pierce, Thermo) with BSA as standards. 10 μg of proteins were separated by 8 or 12% SDS–PAGE, blotted onto a PVDF membrane, probed using protein specific antibodies (Bitoun et al., 2012), and the signals were developed using a SuperSignal West Pico Chemiluminescent kit (Thermo Fisher Scientific) (Bitoun et al., 2012).

### Cell Wall Antigen Preparation and Slot Blot Analysis

Cell wall antigens were extracted from murein sacculi using the formamide technique described by Wetherell and Bleweis (Wetherell and Bleiweis, 1975; De et al., 2017). Murein sacculi were prepared by following the protocols of Chan et al. (Chan et al., 2013) from overnight BHI-grown cultures, and equal amounts by weight were suspended in formamide and heated at 180°C for 30 min in an oil bath. The suspensions were then mixed with 2 volumes of 2N HCl and absolute ethanol (1:19 v/v), centrifuged at 350 ×*g* for 20 min, and the supernatants were collected and mixed with 5 volumes of acetone at 4°C for 4 h. The white precipitates were spun down at 350 ×*g* for 20 min. The pellets were dissolved in water and following centrifugation at 21,000 ×*g* for 10 min, the supernatants were dialyzed (MWCO, 3.5 KDa, Thermo Fisher Scientific) at 4°C overnight, and then freeze dried. The antigen extracts were re-suspended in 1 mL of sterile deionized water. For immunoblot analysis, equal amount of the above antigen preps were blotted onto a nitrocellulose membrane using a Slot Blot (Bio-Dot SF, BioRad), and probed with *S. mutans* whole cell antiserum that was generated using inactivated whole cells of the wild-type, UA159 to immunize rabbits (Lampire Biological Laboratories, Inc.) and was adsorbed with live cells of either the wild-type or the *rgpG/brpA/psr* triple mutant (De et al., 2017). Antigen-antibody reactions were detected using SuperSignal West Pico Chemiluminescent substrate (Thermo Fisher Scientific). The signal intensities were further analyzed using Quantity One (BioRad).

### Statistical Analysis

All experiments were repeated at least three separate times, and the results were analyzed using student *t-*test.

## Results

### The *mecA* Gene Is Part of the Polycistronic Operon With *rgpG*

As predicted (Ajdic et al., 2002) (**Figure 1A**), the results of RT-PCR (**Figure 1B**) revealed that *mecA* (SMU.233) in *S. mutans* is co-transcribed as a polycistronic operon with downstream *rgpG* (SMU.234), which encodes the first enzyme of the rhamnose-glucose polymer (RGP) biosynthesis pathway (Yamashita et al., 1999). Interestingly, analysis of the genetic structure revealed that the regions flanking *mecA/rgpG* are highly conserved among oral streptococci, group A and B streptococci (**Figure 1A**). Upstream the *mecA/rgpG* operon are loci for a hypothetical (SMU.231) and an undecaprenyl-diphosphatase (SMU.232, also bacitracin resistance protein), which catalyzes the dephosphorylation of undecaprenyl diphosphate (UPP) and confers resistance to bacitracin (Jalal et al., 2015). Located downstream of the *mecA/rgpG* operon are genes for SufC, SufD, SufS, SufE2, and SufB of the Suf iron-sulfur cluster assembly pathway, respectively. When analyzed, *S. mutans* MecA shows the best similarity with MecA of *Streptococcus* *agalactiae* (67% identity at amino acid level) and the least similarity with *B. subtilis* (at 21% identity) (**Supplementary Table S1**).

### MecA Deficiency Causes Major Growth Defects in *S. mutans*

**FIGURE 2:**
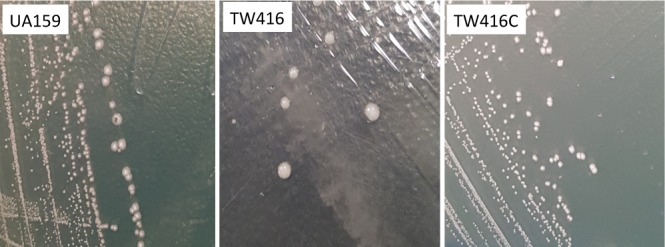
Colony morphology of the *mecA* mutant. *S. mutans* wild-type (UA159), the *mecA* mutant (TW416) and its complement strain (TW416C) were grown on BHI agar plates. Relative to the rough, dry colonies of UA159, the colonies of TW416 were round, mucoid, and smooth. Images were taken using Samsung Galaxy Note V.

**FIGURE 3:**
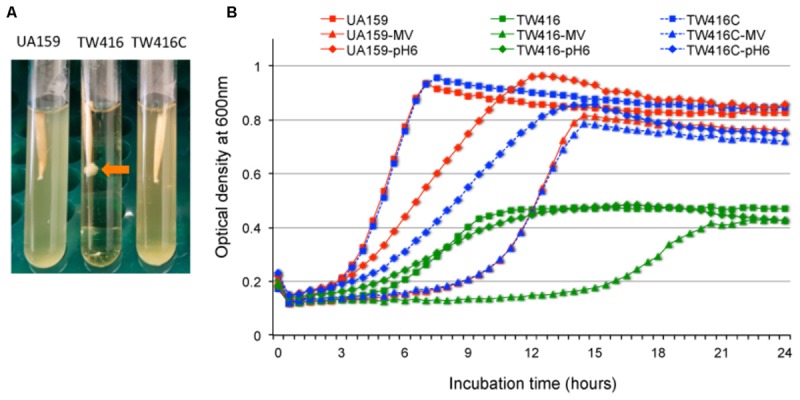
Growth characterization of the *mecA* mutant. **(A)** Growth phenotypes of *S. mutans* wild-type (UA159), the *mecA* mutant (TW416) and its complement strain (TW416C) when grown in BHI broth overnight. Arrow indicates severe clumping of the *mecA* mutant at the tip of the tooth pick. **(B)** *S. mutans* strains grown in BHI broth, BHI broth with pH adjusted to 6.0 and BHI broth with inclusion of methyl viologen at 12.5 mM. The optical densities of the cultures at 600 nm were recorded continuously using a Bioscreen C. Data presented in panel **(B)** are representatives of three separate experiments.

As compared to the wild-type, UA159, the *mecA* mutant, TW416 showed dramatic differences in colony morphology and growth behavior. Unlike the rough, dry colonies of UA159, TW416 formed round, mucoid colonies with a smooth and shining surface (**Figure 2**). Complementation of the mutant in strain TW416C with a wild-type copy of the coding sequence plus its cognate promoter region integrated in the chromosome at the *gtfA* locus in single copy by double crossover homologous recombination restored the phenotypes to the wild-type. Unlike UA159 that tends to grow into the agar medium and becomes hard to pick, TW416 grew spherically on the top of the agar plate and could be easily and completely removed with a single tooth pick. When grown in BHI broth overnight, TW416 clumped severely. Characteristically, it maintained attached to the tip of the tooth picks, as it grew, forming spherical masses, and eventually settled at the bottom of the tubes, leaving a clear medium broth, whereas the parent strain grew in typical homogenous suspension (**Figure 3A**). Similar results were also observed when the coding sequence was replaced with a non-polar kanamycin resistance marker (data not shown). Similarly, complementation of the mutant in strain TW416C restored the phenotypes to the wild-type (**Figure 3A**).

When grown in BHI broth, the mutant had an extended lag phase and an increased doubling time with an average of 3.1 (±0.1) hours, as compared to 1.6 (±0.02) hours for the wild-type (*P* < 0.001) (**Figure 3B**). In addition, the *mecA* mutant also had a reduced final optical density by > 2-fold compared to the parent strain, which can be in part attributed to the severe cell aggregation. Similarly, when grown in BHI broth adjusted to pH 6.0, which is commonly used to test the ability of a bacterium to adapt to low pH environment (Wen and Burne, 2004), the doubling time of the mutant also increased significantly averaging 5.1 (±0.2) h as comparing to 3.8 (±0.08) h for the wild-type (*P* < 0.001) (**Figure 3B**). When incubated in BHI with inclusion of methyl viologen (12.5 mM, final concentration), which induces intracellular oxidative stresses by superoxide radical and hydrogen peroxide production (Wen et al., 2011), the *mecA* deficient mutant displayed an extended lag phase of > 15 h, as compared to 6 h for both the wild type and the complement strain, TW416C.

### MecA Deficiency Causes Severe Defects in Cell Division

**FIGURE 4:**
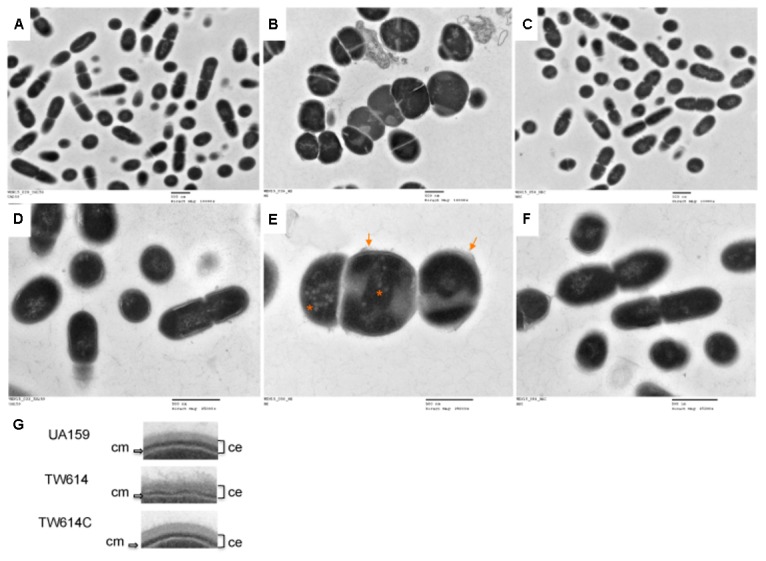
TEM analysis. *S. mutans* wildtype (UA159 and A&D), the *mecA* mutant (TW416 and B&E) and its *mecA* complement strain (TW416C and C&F) were grown in BHI broth until mid-exponential phase (OD_600nm_ ≈0.4). **(A,B,C)** were images taken at magnification of 10,000 × *g*, and **(D,EF)** at 25,000×, respectively. Scale bars represent 500 nm. Images in **(G)** are inserts of blow-up regions of the cell envelope of the different strains with the mutant showing a fuzzy, loose cell envelope (ce) and a thin cytoplasmic membrane (cm), as indicated. The arrows in **(E)** indicate the fuzzy, loose cell envelope and the asterisks indicating low-density patches of the deficient mutant.

Under TEM, UA159 existed primarily as coccoid shaped dividing cells with the septa located toward the end of the cell, whereas the *mecA* mutant, TW416, displayed massive, swollen cells with multiple asymmetric septa (**Figure 4**). A close-up examination further revealed that TW416 had a fuzzy, loose cell envelope and a thin cytoplasmic membrane (**Figures 4E,G**), relative to the compact cell wall of the wild type. Unlike the structured nucleoid of the wild-type, TW416 also had a nucleoid that appeared to be unstructured and contained unusual low electronic density patches (**Figure 4E**). The complement strain TW416C displayed similar morphology as the wild-type, UA159.

### MecA-Deficiency Causes Defects in Biofilm Formation

**FIGURE 5:**
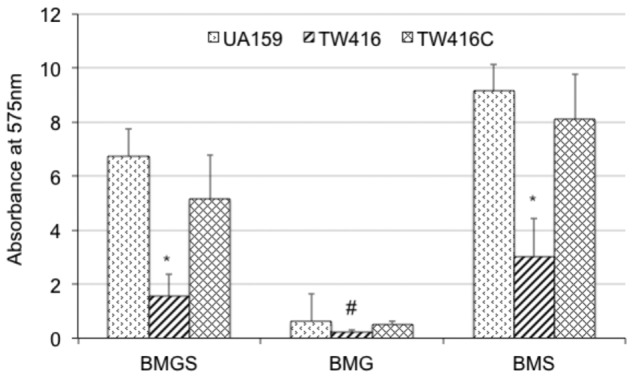
Biofilm formation. *S. mutans* wildtype (UA159), the *mecA* mutant (TW416) and its complement strain (TW416C) were grown in BM medium with glucose and sucrose (BMGS), glucose (BMG) or sucrose (BMS). Biofilms were grown on polystyrene surface in 96 well plates and analyzed using a spectrophotometer. Results presented here represent mean absorbance at 575 nm (±standard deviation in error bars) from three independent experiments and ^∗^ and ^#^*P* < 0.01, *P* < 0.05, respectively.

**FIGURE 6:**
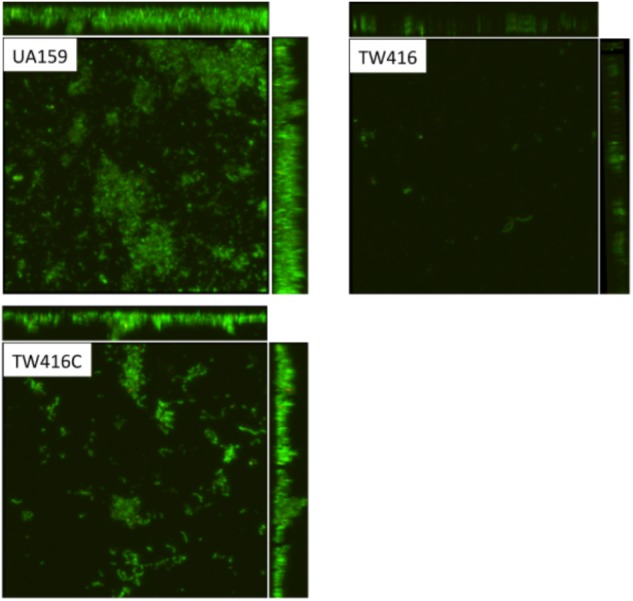
Confocal microscopic analysis of biofilms. *S. mutans* wildtype (UA159), the *mecA* mutant (TW416) and its complement strain (TW416C) were grown in BM medium with glucose and sucrose, glucose or sucrose. Biofilms were grown on HA disks vertically placed in 12 well plates for 24 h, and analyzed using a laser scanning confocal microscope. Panel shows representatives of the compressed confocal images at xy, yz, and xz axis of biofilms of UA159, TW416, and TW416C grown in BM plus glucose and sucrose.

**FIGURE 7:**
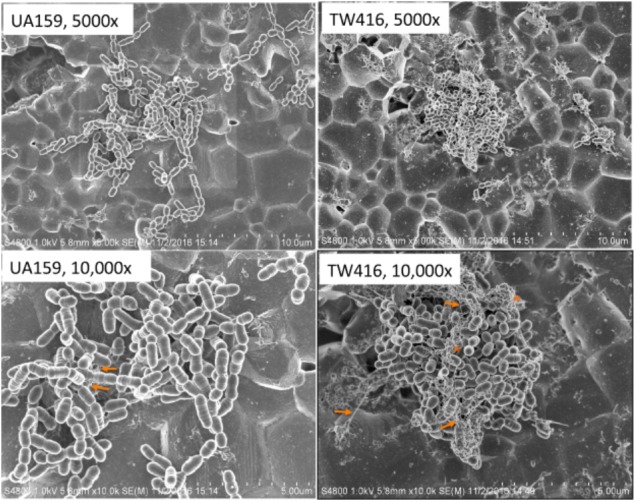
SEM analysis of biofilms. *S. mutans* wildtype (UA159), the *mecA* mutant (TW416) and its complement strain (TW416C) were grown in BM medium with glucose and sucrose, glucose or sucrose. Biofilms were grown on HA disks vertically placed in 12 well plates for 24 h, and analyzed using a scanning electron microscope (SEM). Panel shows images of UA159 and TW416 biofilms grown in BM plus glucose and sucrose, which were taken at magnification of 5,000 and 10,000 × *g* as indicated, with arrows indicating extracellular polymeric substances and asterisks indicating broken cells and cell debris, respectively.

When grown in 96 well plates, TW416 exhibited > 80% reduction in biofilm formation when sucrose was provided as the carbohydrate source (BMS), as compared to UA159 (*P* < 0.001). A similar level of reduction was also observed when the biofilms were grown in glucose alone (BMG, *P* < 0.01) and in the presence of both glucose and sucrose (BMGS) (*P* < 0.001) (**Figure 5**). The complement strain, TW416C, developed biofilms at a level similar to that of UA159 under the conditions tested. To examine the structure and integrity of biofilm formed in absence of *mecA*, biofilms were grown on HA disks and evaluated using confocal laser scanning microscopy (**Figure 6**). COMSTAT analysis of the acquired images showed that UA159 biofilms had an average thickness of 1.1 ± 0.3 μm and a total biovolume of 1.2 (±1.1) μm^-3^/μm^-2^. In contrast, TW416 had a significantly reduced biofilm with an average thickness of 0.04 (±0.02) μm and an average biovolume of 0.03 (±0.01) μm^-3^/μm^-2^. Complementation in TW416C restored biofilm formation with a biovolume of 0.8 (±0.1) μm^-3^/μm^-2^ and average thickness of 1.3 (±0.3) μm. Under SEM, biofilms of the wild-type were featured with evenly spread-out clusters of chained cell, whereas the mutant biofilms appeared dense and clumpy, featuring with the formation of spherical cell masses and the presence of debris of dead cells (**Figure 7**). In comparison, the *mecA* mutant cells also appeared smaller than the wild-type.

### The *mecA* Mutant Is More Resistant to Environmental Stresses

**FIGURE 8:**
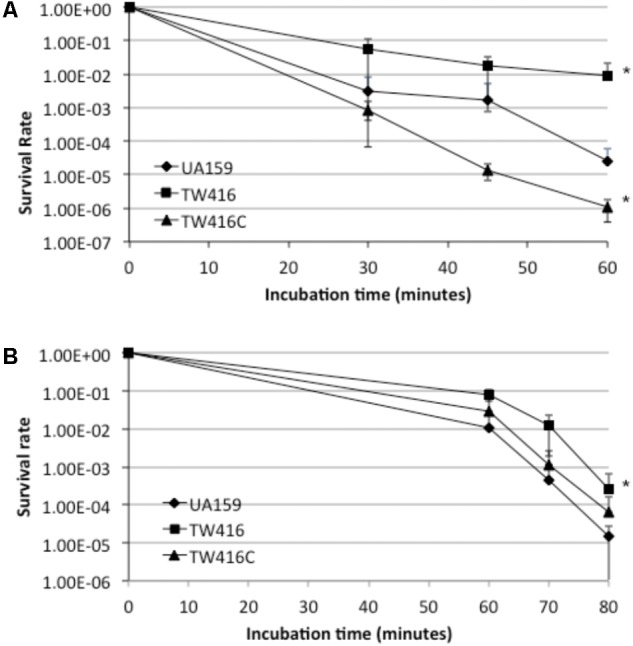
Acid and hydrogen peroxide challenge assays. *S. mutans* strains were grown until mid-exponential phase (OD_600nm_ ≈0.3) and then subjected to an acid or hydrogen peroxide killing. **(A)** shows survival rate of wildtype (UA159), the *mecA* mutant (TW416) and its complement strain (TW416C) following incubation in glycin buffer of pH 2.8, while **(B)** shows survival rate of the strains in the presence of hydrogen peroxide. Data represented here are means (±standard deviation) of at least three independent experiments, with ^∗^*P* < 0.001 as comparing to the wild-type.

Aciduricity and tolerance to oxidative stresses are the two important virulence traits of *S. mutans*. When analyzed for acid tolerance by incubating the bacterial cells in buffer of pH 2.8 for periods of 30, 45, and 60 min, TW416 exhibited an increased level of resistance, as compared to the wild type, with a survival rate almost 2-log higher than the parent strain, UA159 (*P* < 0.001) (**Figure 8A**). Interestingly, the complement strain with a wild-type copy of the coding sequence plus its cognate promoter showed a reduced survival rate than the wild-type. When challenged with hydrogen peroxide for tolerance to oxidative stresses, TW416 also exhibited higher tolerance than the wild-type. After 60 min of incubation, the survival rate of TW416 was > 1-log higher than that of UA159 (*P <* 0.01). Complementation in TW416C restored the phenotype to a level similar to the wild-type, UA159 (**Figure 8B**).

### MecA-Deficiency Increases Susceptibility to Antimicrobials Targeting Cell Envelope

To investigate if MecA deficiency affects the ability of *S. mutans* to withstand stresses induced by cell envelope antimicrobials, the MIC and MBC against lipid II, non-lipid II inhibitors and cell membrane disrupting agents were analyzed. The results showed that relative to the wild-type, the MIC of the MecA-deficient against penicillin G and bacitracin was reduced by 69% for both (*P <* 0.001), by 82% against nisin (*P <* 0.001), by 50% against SDS (*P <* 0.001) (**Table 2**). Slight reduction was observed with Vancomycin, but not with D-cyclone and chlorhexidine (**Table 2**). The MBC of these antibiotics against TW416 were also reduced considerably when compared to the wildtype (**Table 2**).

**Table 2:** Effects of cell envelope antimicrobials on TW416^#^.

Antimicrobial agents	MIC		MBC	
	UA159	TW416	UA159	TW416
Vancomycin	0.75	0.6	1.25	1.2
Nisin	700	125^∗^	1400	250^∗^
Bacitracin	100	31.25^∗^	1600	125^∗^
Penicillin G	0.04	0.0125^∗^	0.075	0.03^∗^
D-cycloserine	350	350	11000	11000
Chlorhexidine	1.5	1.5	6	6
SDS	40	20^∗^	60	37.5

*^#^All concentrations of the antimicrobials used in the study are in micrograms per milliliter*.

*^∗^indicates > 2-fold differences in MIC or MBC as compared to the wild-type*.

### MecA-Deficiency Leads to a Reduced Expression of Known Virulence Attributes

**FIGURE 9:**
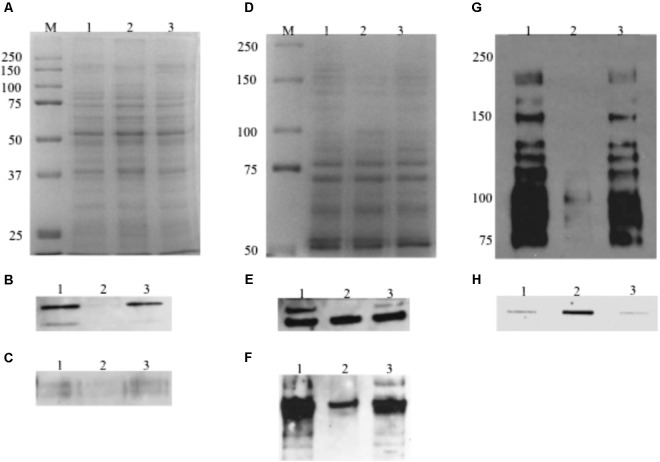
Western blot and slot blot assays. For Western blot analysis of AtlA and WapA, whole cell lysates (10 μg total protein) were separated using 12% SDS–PAGE **(A)** Commassie Blue-stained gel, and following blotting onto a PVDF membrane, probed using polyclonal antibodies against autolysin AtlA **(B)** and WapA **(C)**. For analysis of GtfB, GtfC an SpaP, total proteins (10 μg) were separated using an 8% resolving gel **(D)** Commassie Blue-stained gel and probed using polyclonal antibodies against GtfB **(E)** and GtfC **(F)** and mixture of monoclonal antibodies against SpaP **(G)**. **(H)** For analysis of cell envelope antigens, preps extracted from murein sacculi were blotted onto a nitrocellulose membrane using a Slot Blot and probed using *rgpG*/*brpA*/*psr* triple mutant adsorbed whole cell antiserum. Lanes 1, 2, and 3 represents cell lysate **(A–G)** or cell envelope associated antigen extract **(H)** from the wildtype, TW416, and TW416C, respectively.

When analyzed by Western blot analysis with whole cell lysates and probed with the GtfC, GtfB, SpaP, WapA, and AtlA specific antibodies, the MecA-deficient mutant displayed considerable reduction in signal intensity, when compared to the wild-type and the complement strain. As shown in **Figure 9**, TW416 had little or no immunoreactivity when probed with autolysin AtlA and GtfB specific antibodies, while showed a 73, 93, and 83% reduction in signal intensity when probed with GtfC-, SpaP-, and WapA-specific antibodies, respectively. When luciferase reporter assays were used to examine the *mecA*/*rgpG*, the results showed no significant differences between TW416 and the wild-type, UA159 (data not shown). No significant differences were measured between TW416 and UA159 in luciferase activity when the luciferase reporter gene was fused with the *brpA* promoter and the promoter of the *rgpA/F* operon that encodes genes for the biosynthesis of rhamnose (Shibata et al., 2002) (data not shown).

### MecA-Deficient Mutant Exhibits Higher Level of Cell Wall Associated Antigens

When analyzed by slot blots with the *rgpG/brpA/psr* triple mutant-adsorbed UA159 whole cell antiserum (De et al., 2017), the cell wall antigen extracts from the murein sacculi of the wild-type and the complement strain exhibited similar levels of intensity (350.5 ± 154.5 and 310.5 ± 204.5 INT.mm^2^, respectively) (**Figure 9H**). In comparison, however, the *mecA* mutant was shown to possess 6-fold higher signal intensity than the wild-type (2150.2 ± 459.4 INT.mm^2^, *N* = 3) (*P* < 0.001).

## Discussion

The results presented here have shown that relative to the wild-type, *S. mutans* mutants deficient of MecA displayed severe defects in cell envelope biogenesis /homeostasis, cell division, and alterations in colony and cell morphology. As compared to the wild-type, deficiency of MecA also resulted in enhanced susceptibility to cell envelope antimicrobial agents, but the abilities of the deficient mutants to survive low pH and oxidative stressors were increased significantly. Interestingly, however, the MecA-deficient mutants had reduced cell wall phosphate, whereas displayed an elevated cell wall associated antigens as compared to the parent strain. In addition, the deficient mutant also had a reduced biofilm formation regardless of carbohydrate sources used for growth. These results suggest that MecA in *S. mutans* plays an important role in regulation of cell envelope biogenesis, stress tolerance responses and biofilm formation, traits critical to pathophysiology of this important oral pathogen.

As predicted and proven by reverse transcription-PCR analysis, *mecA* is co-transcribed as part of a polycistronic operon with downstream *rgpG*, which codes for the first enzyme of the biosynthesis pathway for RGP (Yamashita et al., 1999). RGP is a major surface antigen in *S. mutans*, and as shown by our recent studies, plays a critical role in cell envelope biogenesis, cell division and biofilm formation (De et al., 2017). Like MecA, deficiency of RgpG also leads to alterations of cell morphology and reduces biofilm formation. Unlike MecA, however, the RgpG-deficient mutants exist primarily in swollen, giant cells under TEM, whereas mutants deficient of MecA are featured with giant cells with multiple asymmetric septa. Unlike MecA, the RgpG-deficient mutant does not show any major growth defects when growing in BHI broth. In addition, both the spectinomycin- and kanamycin-resistance marker used for construction of the allelic exchange mutants are non-polar, as such *mecA* deletion and its replacement with either spectinomycin- or kanamycin-resistance element should not have any major effects on the expression of *rgpG*. Consistently, complementation of the MecA*-*deficient mutant by double crossover recombination with the *mecA-*coding sequence plus its cognate promoter region of *mecA* also restored all the phenotypes to the wild-type except acid tolerance. When the *mecA* mutant was transformed with pBGK(3)::*PrgpG*, which carries the intact *rgpG* gene plus its cognate promoter region and fully restores the *rgpG* deficiency mutant (De et al., 2017), no such effect was observed in colony morphology and growth defects in BHI broth (data not shown). These results further suggest that the phenotypes observed with TW416 can solely be attributed to the deficiency of MecA.

As the outermost barrier between the cell and the environment, the cell envelope of the Gram-positive bacteria is featured with a thick peptidoglycan and the attached anionic polymers such as teichoic acids and capsules. It is known to play a crucial role in an array of biological processes vital for bacterial pathophysiology, including environmental sensing and signal transduction, maintenance of osmotic pressure, cell shape and size, and cell division. *S. mutans* is not known to possess copious cell wall teichoic acids, and key members of the biosynthesis pathway have not yet been identified (De et al., 2017). However, our recent studies have generated evidence that the bacterium also produces phosphate containing polymers besides RGP and whose attachment to the cell wall are catalyzed by BrpA and Psr, two paralogs of the LytR/CpsA/Psr family (LCP) proteins (De et al., 2017; Wen et al., 2018). Morphologically, the MecA-deficient mutants resembles the *rgpG*/*brpA*/*psr* triple mutants, which also feature giant cells and multiple asymmetric septa (De et al., 2017). Presumably, allelic exchange mutagenesis of *rgpG* and the two *lcp* genes resulted in major defects in polymers synthesis (RGP and others via RgpG) and attachment (the other P-containing polymers) and thus deficiency of cell wall associated anionic polymers. In addition, our recent studies also suggest that the LCP proteins in *S. mutans* may play a direct role in cell division (De et al., 2017). Nevertheless, defects in cell envelope biogenesis /homeostasis are likely part of the factors that underlie the exacerbating susceptibility of the MecA-deficient mutants to cell envelope stressors, including both lipid II (nisin and bacitracin) and non-lipid II inhibitors (penicillin G) and membrane detergent SDS. On the other hand, passive acid efflux and active proton translocation via F-ATPase are two of the primary mechanisms responsible for the survival of the bacterium in acidic environments (Lemos and Burne, 2008). As indicated under TEM, MecA-deficiency in *S. mutans* led to some major alterations in cell morphology, including reduction in cell cytoplasmic membrane. The increased susceptibility of the deficient mutant to SDS, a membrane detergent, also suggests defects in cytoplasmic membrane. It is possible that such membrane defects enhance the permeability of the membrane to weak acids, resulting in more acid efflux and thus increased resistance to acids. However, it awaits further investigation if MecA-deficiency also affects the membrane lipids composition, which is also known to be critical to acid tolerance in *S. mutans’* acid tolerance (Fozo et al., 2007). It remains unknown if MecA-deficiency affects the expression of F-ATPase and the adaptive acid tolerance response. Annotated as *uppP*, the gene immediately upstream *mecA* codes for a undecaprenyl-diphosphatase that catalyzes the recycling of undecaprenyl phosphate, a lipid carrier that is required for the biosynthesis of peptidoglycan and a variety of surface polymers, such as teichoic acid and RGP. *S*. *mutans* strains with deletion of *uppP* was also shown to have a weakened tolerance to bacitracin and reduced biofilm formation, although unlike the *mecA* mutant, no major growth defects are observed with the *uppP* deficient mutants (Jalal et al., 2015). The *uppP* gene is transcribed in the same orientation as the *mecA/rgpG* operon, thus polar effect does not apply. However, it awaits further investigation, if MecA-deficiency affects the expression of UppP, thus the availability of lipid carrier and biogenesis of cell envelope and the related functions including cell division.

Biofilm formation is a sequential process which is initiated with adherence of the bacterial cells to a surface, followed by multiplication and formation of micro-colonies and eventually development of a community embedded in an extracellular matrix. The reduced growth rate, as a result of defects in cell division, is likely a major factor that underlies the mutants’ severe defects in biofilm formation, which appears to be more related to biofilm accumulation rather than biofilm initiation regardless of the carbohydrate sources used for growth. As evidenced by TEM analysis, the cell wall antigen analysis and the cell wall phosphate assay, MecA-deficient mutant also possesses characteristics of cell envelope disturbance. This in turn will also have some major impact on bacterial surface adherence and bacterial cell-cell interactions in biofilm accumulation. Quorum sensing, including the one regulated by the Com system, is also known to play a role in *S. mutans* stress tolerance response and biofilm formation (Li et al., 2001, 2002). However, considering the fact that deficiency of MecA prolongs SigX stability, it remains unclear how the reduced biofilm formation by the *mecA* mutant can be attributed to prolonged competence. In addition, as shown by Western blotting, several proteins known to play critical roles in biofilm formation were also found to be differentially expressed in response to MecA-deficiency. Among the down regulated proteins were the glucosyltransferases GtfB and GtfC. Gtf enzymes produce adhesive glucose polymers, also known as glucans or mutans, which serve as scaffold playing a critical role in biofilm stability and biofilm development. Besides, the Gtf proteins, especially GtfB can also function as adhesins, which bind to glucans and the surface of other bacteria and fungi (Gregoire et al., 2011; De et al., 2017). P1, also known as SpaP, is a multi-functional high affinity adhesin by which the bacterium adheres to the salivary pellicle on the tooth surface (Crowley et al., 1999). Recently, P1 has also been shown to form amyloid protein fibrils, which along with the other matrices, play a critical role in biofilm integrity and stability (Besingi et al., 2017). When probed by a mix of four monoclonal antibodies that recognize different regions of the P1 antigen, the MecA-deficient mutant displayed major reductions in the level of P1 protein in whole cell lysates, although it remains unclear how it influences the translocation and conformation on the cell envelope. Nevertheless, reduction of P1 has been shown to have a negative impact on biofilm formation, stability and development (Lee et al., 1988). WapA, a cell surface associated protein, is well known to have a major impact on biofilm formation and cell surface structure maintenance in *S. mutans* (Zhu et al., 2006). Like P1, WapA also forms amyloids (Besingi et al., 2017). AtlA is an autolysin whose deficiency has been shown to lead to decreases in autolysis, longer chain length, and drastically reduce biofilm formation regardless of the carbohydrate used for growth (Brown et al., 2005). Relative to the wild-type, the level of AtlA is barely detectable indicative major reduction in response to MecA-deficiency, likely contributing to observed defects in cell division and biofilm formation. When analyzed, however, the *mecA* mutant was shown to have an elevated autolysis, when compared to the parent and its complement strains (**Supplementary Figure S1**). Likely, this result can be in part attributed to the defects in cell envelope biogenesis rather than the altered expression of AtlA.

As an adaptor protein, MecA is known to form complex with ATPase ClpC, which recognizes and unfolds specific substrate proteins and translocates them to protease ClpP for degradation (Persuh et al., 1999; Wang et al., 2011; Liu et al., 2013). The capacity to maintain cellular protein homeostasis, commonly termed regulated proteolysis, is central for bacterial pathophysiology (Joshi and Chien, 2016; Bittner et al., 2017; Kuhlmann and Chien, 2017). In *S. mutans*, deficiency of MecA, ClpC, or ClpP has been shown to result in cellular accumulation of SigX and a prolonged competence state, while over expression of MecA enhances proteolysis of SigX and accelerates the escape from competence (Tian et al., 2013; Dong et al., 2014). Theoretically, with the deficiency of MecA, the ClpC/P proteolysis machinery fails to function properly, which leads to disturbance of cellular functions contributing to the altered cell morphology, the defects in cell division and the reduced cell envelope stress and other related phenotypes. On the other hand, recent studies have also shown that the ClpP protease in *S. mutans* affects stress tolerance responses and biofilm formation and regulates large groups of genes including those related to biofilm formation (Lemos and Burne, 2002; Kajfasz et al., 2009, 2011; Chattoraj et al., 2010; Tao and Biswas, 2015; Jana et al., 2016). However, none of the Clp mutants display any phenotypes featured with the MecA-deficient mutants (Lemos and Burne, 2002; Kajfasz et al., 2009; Chattoraj et al., 2010; Kajfasz et al., 2011). Like MecA, deficiency of ClpP was shown to lead to aggregation, reduce growth rate, especially in acid pH, and enhance resistance to acid killing. The ClpP mutant also formed less biofilm when growing in glucose, but unlike the *mecA* mutant, the ClpP mutant increased biofilm formation when grown in sucrose (Kajfasz et al., 2009). Consistently, ClpP-deficiency also caused up- and down-regulation of large groups of genes, including 4-fold increases of GtfB, C, and D (Kajfasz et al., 2011), which is again different from what were observed with the MecA-deficient mutant. Similar results have also recently been observed in *B. subtilis* concerning its regulation of sporulation, exopolysaccharide production and biofilm formation (Prepiak et al., 2011). *In vitro*, MecA interacts with Spo0A, a master regulator of biofilm formation and sporulation, but unlike in competence regulation, such interactions sequester Spo0A from its activity but not for degradation. Recent studies have provided further evidence that MecA, along with its partner ClpC, can directly bind to phosphorylated Spo0A on target promoters, preventing the activation of gene transcription (Tanner et al., 2018). Similarly, the results presented here also suggest MecA in *S. mutans* is more than just an adaptor protein and that factors other than ClpC/P, including regulatory factors involved in cell envelope biogenesis, are likely involved either directly or indirectly in the related functions.

In summary, our study has shown that MecA is an integral factor in regulation of *S. mutans’* pathophysiology. Deficiency of MecA leads to major defects in cell envelope biogenesis, cell division, growth, and biofilm formation. The results presented here also suggest that MecA in *S. mutans* is more than an adaptor protein in regulated proteolysis, although how MecA modulates the related functions awaits further investigation.

## Author Contributions

ZW conceived the experiments. AD, AJ, WB, and ZW conducted the experiments and analyzed the data. AD, AJ, JL, and ZW wrote the manuscript.

## Conflict of Interest Statement

The authors declare that the research was conducted in the absence of any commercial or financial relationships that could be construed as a potential conflict of interest.
