# Supplementary material for: Deficiency of MecA in Streptococcus mutans Causes Major Defects in Cell Envelope Biogenesis, Cell Division, and Biofilm Formation
Source: Front Microbiol. 2018 Sep 11;9:2130. doi: 10.3389/fmicb.2018.02130 (PMC6141683; doi:10.3389/fmicb.2018.02130)
Supplement: Supplementary file 1 [file Presentation_1.pdf]

Deficiency of MecA in *Streptococcus mutans* Causes Major Defects in Cell Envelope Biogenesis, Cell Division, and Biofilm Formation

Arpan De[1#], Ashton N. Jorgensen[1], Wandy L. Beatty[4], Jose Lemos[5], and Zezhang T. Wen*[1,2,3]

[1]Department of Comprehensive Dentistry and Biomaterials, [2]Center of Oral and Craniofacial Biology, and [3]Department of Microbiology, Immunology and Parasitology, Louisiana State University Health Sciences Center, New Orleans, LA, USA; [4]Washington University Department of Molecular Microbiology, St. Louis, MO, USA; and Department of Oral Biology, University of Florida, Gainesville, FL, USA.

Effects of *mecA* deficiency on autolysis

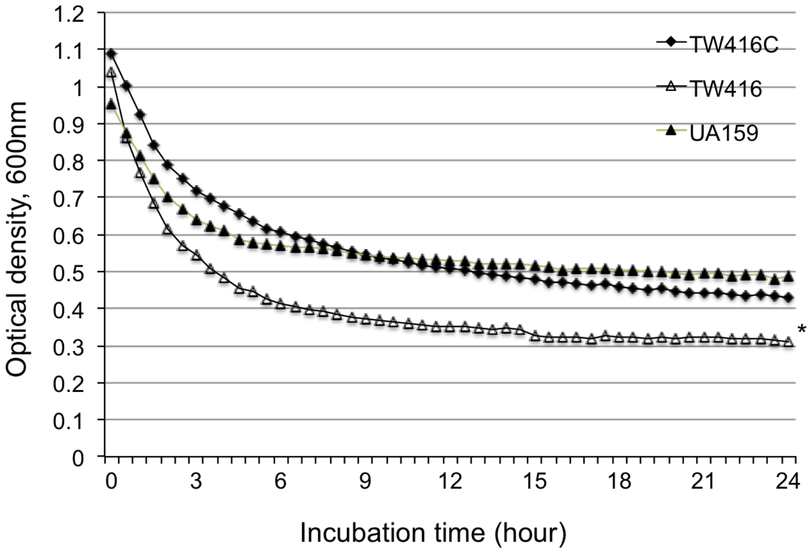

Figure S1. Autolysis assay. Overnight cultures of *S. mutans* wild-type (UA159, solid triangles), the *mecA* mutant (TW416, open triangles), and its complement strain (TW416C, diamonds) were washed and resuspended in phosphate buffered saline, pH 7.2, and the optical density of these cells was then monitored using Bioscreen C. Relative to wild-type UA159, *mecA* mutant TW416 displayed a significantly quicker and more reduction of the optical density over the time of incubation (indicated by an asterisk, $P<0.01$).
